# ‘*Are We Sure That He Knew That You Don’t Want to Have Sex?*’: Discursive Constructions of the Suspect in Police Interviews with Rape Complainants

**DOI:** 10.3390/bs14090837

**Published:** 2024-09-18

**Authors:** Megan Hermolle, Alexandra Kent, Abigail J. Locke, Samantha J. Andrews

**Affiliations:** 1 School of Psychology, Keele University, Staffordshire ST5 5BG, UK; a.kent@keele.ac.uk (A.K.); a.j.locke@keele.ac.uk (A.J.L.); s.j.andrews1@keele.ac.uk (S.J.A.); 2 Institute for Social Justice and Crime, University of Suffolk, Ipswich IP4 1QJ, UK

**Keywords:** policing, rape, investigative interviewing, rape myths

## Abstract

Recent statistics reveal alarming flaws in the Criminal Justice System’s (CJS) handling of rape cases, undermining the pursuit of justice for complainants seeking legal redress. This paper takes a novel approach to explore police rape stereotype use in interviews with rape complainants, utilising critical discourse analysis and conversation analysis and discursive psychology to understand and critique the balance of power within an interview and how this might impact attrition and prosecution decisions. Ten police interviews with rape complainants were analysed with several suspect discursive constructions present throughout, including the interviewer constructing the suspect as misunderstanding, the complainant as miscommunicating non-consent, or agentless and passive talk. A significant and original finding was the way constructions interacted with the spectrum of stranger-to-partner rapes. In stranger rape cases, passive language often obscures the suspect and emphasises the complainant’s behaviour. Acquaintance rapes frequently involved misunderstandings centred on visible distress and mixed signals. Partner rapes highlighted issues around consent and coercion, with officers often ignorant of coercive control and domestic abuse. These findings align with Operation Bluestone Soteria (OSB); thus, the recommendations align with those made by OSB’s Pillar One.

## 1. Introduction

### 1.1. Background

Research suggests that the use of rape stereotypes in decision-making in the Criminal Justice System (CJS) contributes to the widening gap between rapes, their reports and prosecutions [1]. For example, the End Violence Against Women (EVAW) Coalition (2019) [2] found that the Crown Prosecution Service (CPS) had a risk-averse policy, only appearing to take on ‘easy cases’. These contained features which are more likely to fit juries’ social representations of real rape—including stereotypes in which the suspect was a stranger or ‘other’ in some way, physically violent, or the rape occurred outside at night. Consequently, many rape complainants slip through the cracks, as 84.7% of complainants know their rapists and so do not fit real rapist or real rape stereotypes [3]. Munro and Kelly (2009) [4] coined the term ‘vicious cycle of attrition’ arising from this policy, in which prosecutors are more likely to advance cases they believe have a realistic chance of securing conviction. They anticipate jury decision-making, relying on lay stereotype usage that influences this—for example, research has found that juries’ biases and personal characteristics influence verdict decisions, including rape myth acceptance (RMA), which has been found to consistently influence complainant and defendant believability, and both pre- and post-deliberation decisions [5,6]. Police are also more likely to advance cases that conform to ‘real rape’ stereotypes, and the initial evidence-gathering interview with the complainant, which is often pivotal to a case due to scarcity of witnesses and other evidence [7], potentially reflects this stereotype use, highlighting a need for research at this attrition point, as there can be negative impacts in terms of re-traumatisation, feeling shame and internalising blame, and physical manifestations of trauma [8].

Further complicating these challenges is evidence that legal professionals use rape stereotypes without belief in them—King et al. (2024) [9] found that there was a disconnect between what lawyers understood in theory and a reliance on rape stereotypes. This likely reflects wider societal and institutional structures that perpetuate these stereotypes, such as patriarchy and existent power structures [10]. For example, male criminal justice students held higher levels of patriarchal and conservative beliefs [11]. Murphy and Hine (2019) [12] found that certain attitudinal variables, such as hostility towards women and the relationship between power and sex, were significantly predictive of stereotype acceptance. This suggests that stereotypes are being used in conjunction with patriarchal attitudes and placed in context with Munro and Kelly’s (2009) [4] observations and findings by King et al. (2024) [9], used as a decision-making shortcut. Thus, while beliefs may be changing, the use of stereotypes for the purposes of investigation and defence is still problematic, as they are live features of discourse around sexual assault and negatively impact the complainant.

Efforts are additionally underway to shift the focus from the rape complainant to the suspect in the CJS. Operation Soteria Bluestone [OSB] is a large-scale police-academic collaboration launched in 2021 by the Home Office. The Year One report [13] provides a wealth of findings across six different pillars of research. Pillar One reports that investigations were disproportionately complainant-focused, and complainants had to prove credibility and integrity [14]. Social representations of rape, reflective of real rape stereotypes, often drove investigations and interviews. The researchers concluded that the suspect’s behaviour and choices should be the focus of rape investigations [13]. A full account of the events does need to be elicited from the complainant in addition to this, so some sensitive questions must be asked—however, they are often asked in a way that causes re-traumatisation or feelings of isolation and alienation or no explanation is given as to why specific lines of questioning are being used [15].

### 1.2. Rape Attrition

The CJS faces many challenges in mitigating the cycle of attrition. Attrition rates appear highest at the police interview stage—in addition to the rapes that go unreported, Daly and Bouhours’ (2010) [16] review of attrition found that across multiple countries, only 30% on average of reported sexual offences resulted in a charge, while Gillen (2019) [17] noted that in Northern Ireland, 40% of rape complainants later withdrew their allegations. Hohl and Stanko (2015) [18] found that rape complainants withdrew from the process for several reasons, including feeling re-victimised and disbelieved and a lack of faith in the CJS. Recent research has similar findings around the barriers to reporting [19], including the same lack of faith, which involved a perceived lack of evidence and the traumatisation of reporting; self-blame; and knowing the suspect, suggesting that similar barriers to reporting face those who do report and then withdraw. Attrition rates are also significant at other key stages of the process—Willmott et al. (2021) [20] noted that prosecutors often make charging decisions based on how a jury will interpret the facts. Those cases that do go to trial are unlikely to be convicted: where 55,130 allegations of rape were recorded in 2019–2020 [3], just 3.8% were prosecuted and 2.6% resulted in a conviction [21].

### 1.3. Discursive Features of the Police Interview

Due to the high levels of complainant withdrawal at the police interview stage, the current study focuses on the initial evidence-gathering interview with rape complainants. Critical Discourse Analysis (CDA) was chosen for the macro analytic approach to the study because it examines how language is used to display ideologies and power structures in investigative interviews, highlighting potential issues with questioning techniques [22]. Gender- and rape-related ideologies can be compared to social representations of rape and violence against women, resulting in stereotypes.

Police interviews can provide valuable insight into the challenges facing the CJS when interviewing rape complainants. One notable feature of interviews is their example of institutional discourse. Participants’ talk in institutional settings is influenced by the interaction between their interactional and discursive role (i.e., as interviewee) and their institutional status (i.e., as complainant or witness). There is an inherent power asymmetry between the interviewee and the interviewer. Haworth (2006) [23] pointed out that interviewers hold institutional power and are empowered to make crucial decisions whose outcomes affect the interviewees’ futures. Additionally, the police interview is not an isolated discursive event but part of a wider process—while it is an instrument for evidence gathering [24], it is also evidence in itself. Thus, not only is the interview for the benefit of the police interviewer to aid decision-making but also for an overhearing audience [25]. When real rape and real rapist stereotypes are used in interviews, they are subsequently available to be presented by lawyers to juries. MacLeod (2010) [26] found evidence of stereotype use in police interviews with rape complainants, finding that some discursive features, such as formulations (rewording immediately prior to talking), functioned to clarify details for the overhearing audience. However, the complainant’s behaviour was foregrounded while the suspect’s was backgrounded. Antaki et al. (2015) [27] showed that police interviewers built up a pattern of accountability, asking conduct-related questions (‘How come you didn’t...?’; ‘Why did/didn’t you...?’). Although police interviewers aim to test alternate theories, these questions reflect complainant-blaming stereotypes and real rape stereotypes (when the conduct in question is related to struggle or visible distress). This distressed the complainants, and research indicates that why/how-come questions can imply doubt and disbelief and that there is no adequate account when the asker is in a position of epistemic strength [28]. This can intensify feelings of self-blame. The current study builds on these insights to investigate the asymmetry of police interviews more directly than has been performed previously.

### 1.4. Current Study

Using a novel methodologically pluralistic approach to the analysis, we explored police rape stereotypes used in interviews with rape complainants using conversation analysis, discursive psychology, and a critical discourse perspective to understand and critique the balance of power within an interview, and how this might impact attrition and prosecution decisions building on previous similar research [24,25,26,27]. The highest attrition rates for allegations of rape are during the initial police investigation stages. Taken with the evidence of higher acceptance of ‘real rapist’ or suspect stereotypes amongst legal and policing professionals [29,30,31,32], there is an urgent need to explore rape stereotypes in police-complainant interviews. Two main discursive constructions are discussed in the current study through a CDA perspective of power and ideology: miscommunication and passive and agentless talk. This section outlines some definitions and literature.

The miscommunication model of rape proposes that acquaintance rape is the result of miscommunications and misunderstandings [33]. This has been criticised: men have been found to understand a variety of sexual refusals despite claiming to misunderstand indirect refusals, while women have found it difficult to refuse sex directly [34,35]. Similarly, Marcantonio et al. (2018) [36] found in a survey on sexual communication that women used a variety of sexual refusals, while Beres et al.’s (2014) [37] thematic analysis noted that participants did not rely on miscommunication stereotypes to resolve ambiguous sexual situations.

Ehrlich (2003) [38] coined the term ‘agentless passives’, positioning grammatical choices as ‘potentially important social acts’. She pointed out that when suspects and legal professionals utilise them, it diminishes perceived responsibility and shifts blame onto the complainant. This usage has been observed recently—a thematic analysis of rape coverage in UK newspapers identified the broad use of passive terms and agentless grammar, obscuring the suspect [39]. Our use of ‘agentless passives’ or ‘passive voice’ throughout aligns with Ehrlich’s understanding and definition [38].

Each of these discursive constructions has the effect of obscuring the Mens Rea element of rape through deleting or diminishing agency and backgrounding and exonerating the suspect. Mens Rea is defined by the Sexual Offences Act (2003) [40] as the suspect having ‘no reasonable belief of consent’. It is often easy to prove the Actus Reus element of rape—the intentional penetration of the vagina, anus or mouth of another person with his penis without consent—but harder to prove Mens Rea. This is potentially why constructions which diminish any suggestion of ‘no reasonable belief of consent’ are used. This study takes its definition of rape from [40], which defines rape as intentionally penetrating the vagina, anus, or mouth of another person with a penis without the other person’s consent, and when the perpetrator does not reasonably believe that the victim consents. This allows some space for contemporary understandings such as affirmative models of consent, which can involve verbal or nonverbal cues—‘yes means yes’ or nonverbal non/consent [34,41]; however, it primarily follows the traditional ‘no means no’ and implied consent model, which the CJS works from. The data utilised in this study were from 2015–2017, meaning that institutional attitudes and conversations may have evolved in the meantime; however, Operation Soteria Bluestone’s (2024) findings were broadly that by 2021–22, there was still a focus on communicating non-consent and ‘no means no’, with some isolated acknowledgements of coercive control-based unwilling consent [9].

Based on previous findings indicating that perpetrator stereotypes are more likely to be utilised than any other type [42], these stereotypes were explored for the present research to answer the following questions:

- How are suspects constructed within the interviews?
  o How do these constructions differ from the different relationships between complainant and suspect (i.e., stranger, acquaintance, partner)?
  o How are these construction formulations responded to by the complainant?

## 2. Materials and Methods

### 2.1. Data and Preliminary Analyses

Ten real-life initial evidence gathering video recorded police interviews with female rape complainants whose suspects were male, conducted in the West Midlands of England in accordance with Achieving Best Evidence (ABE) Guidelines [43], were identified, giving 10 h and 22 min of data that were analysed overall. The interviews all conformed to the guidelines at a top-level reading, while during the closer analyses, the first author found occasions where the interviewer diverged slightly, showing the benefits of the pluralistic methodological approach. The complainants were all women and all White, aged 18 to 45 years. Three cases were stranger rapes, four were acquaintance rapes—ranging from less well-known to close friend—and three were partner rapes. Three interviewers were male, while seven were female. Some interviews were conducted in a Sexual Assault Referral Centre (SARC), and the others in a police station. The interviews were recorded between 2015 and 2017 by one police force and lasted between 24 min and 1 h and 55 min.

The interviews were transcribed verbatim, and the first author closely read the verbatim transcripts to detect examples of rape stereotypes. Utilising the rape stereotype scale from Hermolle (2023) [42], the most common rape stereotype themes within each interview were identified. All potentially relevant moments from each interview were analysed across multiple successive rounds of iterative analysis. One transcript, a stranger rape, was eliminated on the basis that the complainant’s statement would not likely have been used as primary evidence because the interview did not contain any rape myths and because the case was very different to the others, as there was video evidence and the complainant had little memory of the event itself. While some other stereotype use was found within the interviews, suspect-related stereotypes were the most consistent and widespread across the transcripts. The research questions were developed from these.

Salient extracts were then identified for Jefferson transcription [44] to explore nuance within the constructions, especially in terms of emphasis, tone, and picking up distress or emotion not evident in the spoken word. Twenty-five extracts were identified from all relevant cases and subject to close Jefferson transcription, with six representative examples utilised in the final study. The analysis was continuous and cyclical throughout the process from the first step. It started inclusively, becoming more specific and precise during the systematic exploration of the cases and, eventually, extracts.

### 2.2. Theory and Methodology: An Integrated Approach

To comprehensively explore police interview features and how they affect the participants and reveal interviewers’ and institutions’ social representations of rape, an integrated, multi-perspective and bottom-up approach to the data was used. Consequently, some micro-level features were drawn from conversation analysis and discursive psychology. For example, interruptions [45], reported speech [26], the use of justifications [46], and interpretative repertoires in regard to reliance on neutralising language and ‘appropriate resistance’ talk [47,48] were analysed in combination with a macro-level analysis: the socio-cognitive Critical Discourse Analysis (CDA). This placed the micro-level features in the wider context of power and asymmetry, disbelief and doubt, and social representations of rape reflected within the institution and in individual interviewers. MacLeod (2010) [26] conducted a comparable CDA on a similar sample using this approach, while van Dijk (2001) [49] suggested that a CDA should be diverse and multidisciplinary, integrating the ‘best work of many people’ and that CDA should involve examination of interactional control and interactional content, which lends credence to the current study’s novel, multi-perspective approach.

An integrated discursive approach helps us to understand individual meaning-making (Jefferson-level analysis), relational dialogue (what business is happening in the interview and what the effects are on the complainant), and what power relations are happening at the institution level, which is also affecting the interview (i.e., pressures on the interviewer to ask inappropriate questions which could be asked in court). This allows further understanding of the negotiation, resistance, and perpetuation of social representations of rape within the police interview and the wider police and CJS culture.

### 2.3. Defining Complainant-Suspect Relationships

Due to the differences in misunderstanding constructions and use of agentless passives depending on the complainant–suspect relationship, this study analysed the findings across a spectrum of suspect acquaintanceship, from ‘stranger’ to ‘partner’. Thus, it is necessary to establish a working definition of a stranger rape, an acquaintance rape, and a partner rape, to understand which category each extract falls into (see Table 1).

## 3. Results

This section explores the constructions of the suspect along a spectrum of alleged stranger rape to acquaintance rape. This structure is useful for the analysis because the data were unusually striking in how strong the alignment was between the type of discursive constructions used and the complainant-suspect relationship. The less stereotypical the rape, i.e., the more the complainant and suspect knew each other, the more complex the questioning became. In partner rapes, the focus was typically on the suspect’s understanding of consent, coercion versus force (and often, the interviewing officer’s blurring of this distinction), verbal versus nonverbal non-consent, and the ‘rough’ sex narrative. Passive talk consisted of agentless talk and nominatives and increased neutral and non-violent reformulations of violent acts. In acquaintance rapes, misunderstanding constructions recurrently centred on visible signs of distress, lighting at the time of the rape in terms of what the suspect could see, and ‘mixed signals’. Passive talk centred on bodily autonomy and the complainant being ‘done to’ instead of the perpetrator ‘doing’. In stranger rapes, the focus was on agentless and passive talk, with some examples of mistranslation constructions. The more acquainted the complainant and suspect were, the more occasions of misunderstanding and passive talk occurred overall, indicating the presence of ‘real rape’ myths.

In all extracts, IO stands for the interviewing officer, while IE stands for the complainant. INT[number] identifies different interviews. See Table 2 for transcription conventions.

### 3.1. Stranger Profile

This section concerns the constructions of consent misunderstandings within the interviews, including potential miscommunication or the suspect missing the complainant’s nonconsent altogether due to the complainant not properly communicating it.

In Extract 1, the complainant and suspect had first met two hours previous to the suspect following the complainant to her room. The talk during this extract relates to another person present before the rape, who translated for the complainant and the suspect.

**Extract** **1:Int04:** *So you don’t think there was any miscommunication going on?*


1   IO4: Okay,.hhh How good is his English i’ve spoken to *M1* erm
2        (0.5) her english is uh ((sharp inbreath through teeth))
3        limited i think’s the [best thing to say isn’t it.]
4   IE4:                    [ ***M1***::’s *((female witness))* ] is (.)
5        better than ***C1***’s *((male not present for assault))* and
6       [this ***V***]***1***= *((suspect))*
7   IO4:[right.]
8   IE4: =he- his english was next to no:thing really, 
9   IO4: Oka:y*?*
10   IE4: Erm anything that he did want to say his cousin- er- ***J1*** (0.7)
11        or ***M1*** were translating it for me,
12       (.)
13  IO4: R[ight, ]
14  IE4: [And the]n they were translating what i’d said (.) back to 
15       him,
16  IO4: Were they translating the bit where he’s saying you-you’d-
17       you haven’t >got a< bo:yfriend and everything or(.) was he 
18       say[ing that to you,]
19  IE4:   [No, he-he ] can s- he can speak some e:nglish, but
20       (0.8) for somebody that (1.7) is not very good with accents
21       it’d be very hard for them to understand,
22  IO4: °Right°,=
23  IE4: =Erm (0.4) i (.) i only pick up some bits cause of (0.2) how
24       ***M1*** talks to me and what she says to me, 
25  IO4: Yeah.
26  (.)
27  IE4: Erm (.) so that’s the only reason that i pick them up but 
28       when he was trying tell me i was beautiful (0.4) and i’d got 
29       nice eyes he didn’t know how to say tha:t (.)
30       in [engli]sh, (0.3) So then ***J1*** was=
31  IO4:    [Yeah,]
32  IE4: =translating things like that_ 
33  IO4: Right.
34  IE4: And then when i was saying i’d got a boyfriend and i was
35       happy ***J1*** was translating that to him to let him know what i
36       was saying, 
37  IO4: Oka:y, .hhh So (.) here’s the thing then i >mean i-< (0.9) i
38       don’t speak Slovakian, i don’t suppose(0.2)
39       you do e:ith[er. D’you TH]Ink *J1=*
40  IE4:             [(hh)No(h)o. ]
41  IO4: =was translating the correct things to you did you get the
42       impression that ***J1*** was telling him
43       (.) [the correct things_ ]
44  IE4: Yeah [because he was saying so]me of the things in E:nglish
45       as w[ell, ]
46  IO4:     [Right.]
47  IE4: So i knew what- i kinda knew what he was sa:ying,
48  IO4: Yep,
49  IE4: Cause he’d say it in his language and then he’d tell me what
50       he’d just said to him in english and so would ***M1***.
51  IO4: .hhh Right, so you don’t think there was any
52       miscommunication [going on.]
53  IE4:                  [No ]definitely not, cause ***M1***, she
54       .hhh (0.7) sh-she’d tell ‘em straight really she’d tell them
55       that i didn’t say that o:r what i did say (.) d’you know what
56       i mean, [she’ll-]= 
57  IO4:         [Yeah. ]
58  IE4: =she’ll tell ‘em.


In Extract 1, the interviewer asks multiple questions to set up a misunderstanding construction of the suspect. She first asks, ‘how good is his English’ (line 01), comparing to another non-English speaker who was present. After several turns (lines 4–35) in which the interlocutors attempt to reach agreement on the level of English fluency, the interviewer then begins a so-prefaced question (lines 37–43), signalling the end of the attempt to agree on the ‘facts’ of the language ability and a shift to deliver the upshot of her original question about the suspect’s English ability. She breaks off from the question with a turn-medial parenthetical insert [53]: ‘Here’s the thing then I mean I (0.9) I don’t speak Slovakian, I don’t suppose you do either’ (lines 37–38). This insertion enables the interviewer to shift from an institutional footing to a more interpersonal one and creates a moment of shared alignment with the complainant, offering acceptance and solidarity about not speaking Slovakian. We know, from earlier in the interview, that the interviewer has already established that the complainant does not speak Slovakian, so when it is raised here, it is not as a neutral information solicit but in service to the relational move. The complainant’s confirmation of shared non-Slovakian speaking contains interpolated laughter particles that also mark the moment of shared alignment. The interviewer then resumes the so-prefaced question with ‘do you think J1 was translating the correct things to you’ (lines 39–43), which directly introduces the possibility of miscommunication.

The interviewer’s footing shift towards conversationalisation [54] and relatability on lines 37–38 is in line with the Enhanced Cognitive Interview training, which recommends interpersonal communication [55]. By using it in this specific sequential context, it functions to make it interactionally easier for the complainant to admit to language difficulties and confusions during the assault by establishing the reasonableness and common likelihood that anyone in her position (including the interviewer) would have struggled to communicate. On both an interactional and an ideological level, the attempt to create interpersonal solidarity conveys the interviewer’s personal presumption of miscommunication but also the ideological presumption of the wider police institution and CJS context that she acts as a representative for in the interview.

Although the question is grammatically formatted with an interactional preference [56] for an agreement that the translation was correct (‘Do you think J1 was translating the correct things to you’), the shared agreement that they do not speak Slovakian makes it impossible to be confident in a translation. This makes a ‘yes’ response interactionally accountable and more problematic. We can see this impact on the complainant’s response because continuing to resist the ‘miscommunication presumption’ requires her to include an account that negates the need to speak Slovakian because ‘he’d say it in his language and then he’d tell me what he’d just said to him in English and so would M1’ (lines 49–50).

The interviewer immediately upgrades their so-prefaced upshot [57] with the much more explicit, ‘so you don’t think there was any miscommunication going on’ (lines 51–52). Johnson (2002) [58] suggests that so-prefaced questions in police interviews can function to label and evaluate prior utterances and direct the interviewee towards reformulation. They can be used to express dissatisfaction or disbelief with the interviewee’s statement. That appears to be the case here. To agree with this summation would require the complainant to express complete confidence in all aspects of the communication. That is a high bar to achieve, given the multilingual, multi-party nature of the interaction leading up to and during the assault. It would require them to explicitly reject the interviewer’s personal and institutional presumption of miscommunication, thus undermining the shared solidarity. Such formulations are designed to be difficult for recipients to navigate without conceding their position in favour of their interlocutors—namely, that there were communication difficulties during the assault that might reasonably blur nonconsent.

The interviewer’s question (lines 51–52) is designed using the passive voice to avoid attributing responsibility for the miscommunication to a specific agent. However, doing this during a question directed to only one party to the assault positions them (the complainant) as able to answer the question and, therefore, take unilateral responsibility for the subject of the question. Such formulations delete the suspect’s role in miscommunications and background their responsibility for the assault behind the complainant’s role [38]. This is a practice that recurs extensively throughout our dataset. We illustrate this using Extract 2, which comes from a ‘perfect’ stranger rape case—the suspect followed the complainant while walking home at night. The only constructions present in this interview are agentless passives and violence-neutralising language. However, they are used to such an extent that they confuse the complainant.

**Extract** **2:Int07:** *What was that action doing?*


1   IO7: So you know >when you were< talking about your He:ad,=
2   IE7: =Yeah.
3   IO7: .hhh How many ti:mes (0.3) d’you think that (.) your head’s
4        been banging,
5   (3.5)
6   IE7: °say about four ti:mes°,
7   (0.4)
8   IO7: .hh And whe:re has your head been banging,=
9   IE7: =Like twice on my front and twice on my back, .hh Not sure if
10       it was any mo:re I was just (0.6) I think it was about four
11       times,
12  IO7: .hhh Okay, (2.1) AND HOW’S YOUR HEAD BEEN BANGING (.) how has
13       that come to be,
14  (0.4) 
15  IE7: °Mm cause I° wouldn’t let him do anything so (.) like .hhh I
16       remember on my front, he kept like grabbing my ha:ir, (.)
17       ((grabs own back of hair with hand)) 
18  IO7: Yeah,=
19  IE7: =And like was (0.3) like (1.2) just (.) **d**oing that kinda
20       thing ((makes hitting motion with hand)) with my head cause I
21       could feel him gripping my he:ad, 
22  (.) 
23  IO7: Ye:ah,
24  IE7: And then (0.3) >all of a sud<den my face was on the floor,
25       (0.8) So he just was (0.5) I think it was just that kind of
26       (0.3) action kinda thing. ((repeats hitting motion))
27       (0.3) .shIH [>and then-<]
28  IO7:            [ So        ] that action- what’s that action-
29       (.) what was that action do:ing? ((copies hitting motion))
30  (0.9) 
31  IE7: what d’you mean, Like (0.2) ((repeats previous motions))
32       gripping my hair and (0.2) pu[shing me to the] floor_ 
33  IO7:                              [   yeah_       ] (0.6) Yeah_= 
34  IE7: =Yeah. (1) I don’t know how like
35       [explain that,]
36  IO7: [S- and your f]ace where’s-where’s that hi:tting


The interviewer asks a series of questions oriented to a passive agentless grammar—the talk has no actor, and the questions are in the passive voice (lines 3–4, 8, 12–13, 28–29). She asks how many times, where, and how ‘has your head been banging?’ The suspect is not present in any of these formulations, although the complainant stated previously that the suspect carried out this action. This backgrounds the suspect and reduces his role in what happened. Uncertainty is created about who has done what [38], which diffuses responsibility and serves the ideological function of hiding the actor. The interviewer could have asked instead: ‘How many times did he bang your head?’ ‘Where or whereabouts did he bang your head?’ Or, ‘How or why did he bang your head?’ The passive voice is not necessary to avoid leading questions here, as the complainant has already stated it was the suspect who ‘banged’ her head. Including ‘...do you think...’ (e.g., how many times *do you think* he banged your head?’) would have enabled the interview to preserve a neutral stance without making the questions incomprehensibly vague. The persistent use of the passive voice here seems to be an ostentatious way to background and minimise the suspect’s agency in favour of the complainant’s.

The complainant’s first two responses are fitted to the agentless constructions (6, 9–11) suggesting an initial willingness to conform to the institutional norms reproduced through formal interviewing phrasing. However, after the third agentless question (12–13), she reintroduces the suspect’s agency by reaffirming her active non-consent to his actions: ‘I wouldn’t let him do anything’ (line 15) and goes into further detail on how he banged her head on the floor uses active formulations throughout the response. She justifies attributing agency to the suspect through her memory (‘I remember’ 15–16) and physical senses (‘I could feel him gripping my head’ 20–21). Including evidentiary justifications to support attributions of the suspect’s agency in this sequential position displays the claimant’s recognition that the interviewer is creating an interactional context in which direct attributions of the suspect’s agency are unwelcome. She does additional discursive work to ensure they can be included in order to manage the possibility of perceived responsibility or blame when making her non-consent clear.

Later, removing the agent from the talk becomes an obstacle to understanding: The interviewer asks in lines 28–29, ‘So that action- what’s that action (.) what was that action doing?’, regarding the suspect pushing the complainant to the floor. The interviewer’s disfluency highlights the non-normative use of a passive formulation for this type of question, generates confusion, and leads the complainant to seek clarification (line 31). Even with gestural context, Jefferson transcription, and further transcriber clarification, it is difficult to understand what is being asked. There is no true agent in the question due to a nominalisation that presents ‘that action’ as the agent, deleting the suspect from the talk. The complainant’s response—‘Yeah. (1) I don’t know how like explain that’ (lines 34–35)—following delay (line 30) and clarification (lines 31–32) displays continuing trouble with either or both the question’s subject (what happened to her head) or turn design (agentless construction). The difficult and obfuscating nature of the question design exemplifies the interviewer’s discursive power over the complainant. The interviewer chooses how to ask the question, and the interviewee can either align with or resist the question’s implications. Confusingly formulated questions can impact the complainant negatively because it is not clear whether their inability to answer arises from their own unreliable testimony or the interviewer’s unclear interaction. Removing the suspect as a participant in the rape, along with less violent terminology such as ‘hitting, banging’ rather than the complainant’s preferred ‘bashing’ earlier in the interview, allows the overhearing audience to further obscure the Mens Rea of the act. MacLeod (2010) [26] discovered that some interviewers would restate complainants’ words in less violent terms, despite the ABE guidelines [43] recommending that interviewers’ restating of a complainant’s account should be as close to the original words as possible. The interviewer is thus aligning with the ideologies of the institution and wider society, which exonerates suspects through rape stereotypes and social representations [12].

### 3.2. Acquaintance Profile

Moving on to acquaintance rape, misunderstanding constructions become more pointed and personal (e.g., visible distress’) compared to the vaguer miscommunication constructions seen in stranger rape interviews. Extract 3 is from an interview in which the complainant had been to a university event, and the suspect had assaulted her after two days of group socialising. The questioning in Extract 3 is about when the complainant began to visibly cry.

**Extract** **3:Int 10:** *Could he have seen tears in your eyes would you say?*


1   I10: Okay. .hhh (0.7) Did you (0.9) again this is impo:rtant, 
2        (0.2)Did you .hhh (.) have any tears before you said you 
3        started crying (.) in the bed,= 
4   W10: =Yea- (0.2) erm (.) i had (0.3) >sort of< (0.6) it wasn’t (.) 
5        proper tears but my eyes (0.2) i think (0.2) my eyes were 
6        quite watery and sort of (0.7) some tears (.) but .hhh 
7        (0.3) [it was when- ]
8  I10:        [Would you look-] could he-could he have seen .hhh 
9        tears in your eyes (.) would you say, 
10       (.)
11  W10: Erm,
12      (0.5)
13  I10: And y’[ave to be honest about that, ] 
14  W10:       [   I  th-  (0.5)   i  th-     ], °i-i-i think° so erm 
15       (0.4) i-i wuh- i didn’t- couldn’t see my face so (0.2) i’m 
16       not sure but i think so, .hhh [but there’s- ]
17  I10:                               [Were you were] you v- .hh you 
18       know obviously when someone visibly 
19       crie[s you know, D-were you-]=
20  W10:     [ Mhm (0.4) yeah, ]
21  I10: =did you cry .hhh (0.4) you s- you said you cried in the bed 
22       but did you (0.2) did you cry visibly (1) uh-up to that point 
23       at any time,= 
24  W10: =Em (1.4) noh- (1.5) °a l-° (.) lit>tle bit< but not (0.5) 
25       really, (0.2) Erm (0.2) sort of- (0.2) i had like a >couple 
26       of< tears but not (0.3) loads .hhh (0.2) it was- .hhh when he 
27       sort of pinned me down (0.2) and (0.4) i kind of (0.4) had a 
28       realisa:tion, .hhh (0.5) i just (.) sort of (1) i panicked 
29       cause I just thought he was (0.3) bout to (.) rape me >and i 
30       just< (1.4) .hhh (0.2) °°i just°° (0.4) like (0.2) sort of 
31       (0.2) started (.) sort of shakin:g, quite (0.7) drastically 
32       and just (0.6) cryin:g,
33       (0.3)
34  I10: Mkay. .hhh


The interviewer begins setting up a misunderstanding construction when he asks, ‘Did you have any tears before you said you started crying in the bed?’ He uses a reminder of importance—‘did you (0.9) again this is important (0.2)’ (lines 5–7). This reminder is redundant, as from a socio-cognitive perspective, the complainant is aware of how important the interview and getting the facts right is. This is her experience and her account, which she came to talk about. Therefore, while a potential way to seek clarity, in this context, it expresses disbelief and challenges the complainant’s account. An interviewer utterance several turns later: ‘and y’ave to be honest about that’ (line 17) further conveys disbelief in the complainant’s account and draws on the real rape stereotype of ‘visible distress is necessary to communicate clear non-consent’ in line with an older ‘no means no’ model, where the absence of enthusiastic affirmative (nonverbal or verbal) consent is not necessarily considered non-consent, potentially minimising other ways of indicating non-consent [59].

The interviewer holds institutional power in this setting and thus can decide what is relevant in any particular context. By discursively foregrounding the complainant’s visible distress and truthfulness, he also marks it as institutionally more significant than the suspect’s behaviour. This may be a case-building line of questioning for the purposes of corroborating with the suspect later, but as the interviewer has not communicated why he is pressing this point, the effect on the complainant is negative. The complainant pauses and stutters throughout the extract, indicating discomfort and that she may treat the interviewer’s questions as an expression of disbelief. She also challenges the difficulty of putting herself in the suspect’s shoes in response to lines 12–13 (‘Could he have seen tears in your eyes (.) would you say?’): ‘I didn’t-couldn’t see my face so (0.2) I’m not sure but I think so...’. The interviewer interrupts this utterance, ending her turn. This may be a ‘power’ type interruption, as opposed to a ‘rapport’ type [45]. It takes discursive control back from the complainant, asking her if she cried visibly ‘at any point in time’, with the appeal to common knowledge that: ‘you know when someone visibly cries...’ (lines 21–23). The interviewer appeared to orientate to visible crying and distress as a performative act for the suspect to show that she did not consent to avoid misunderstanding. If she had not cried, even performatively, then she had not communicated her non-consent sufficiently. In the wider institutional context, Maddox et al. (2012) [30] found that an ‘appropriate’ amount of distress was more credible to investigators than too much or too little distress, indicating that this interviewer was aligning with the institutional ideologies of the CJS.

Extract 4 is also from interview 10, and while the talk here is mostly active, it still contains a grammar of nonagency. The line of questioning is about the suspect’s body and actions, removing his decisions and mind from the talk.

**Extract** **4:Int 10:** *What was his body doing at that time?*


1   I10: =.hhh How- how many- you know how long was it befo:re (0.2) 
2        his penis entered your mouth_ 
3   W10: Erm (0.5) °there’s° (.) literally straightaway
4        (0.4) °cause° [after] I told him to (0.2) stop=
5   I10:               [Okay,]
6   W10: =and I was >trying to tell him to stop< and get off and_
7   I10: Yeah.
8   (.)
9   W10: As my (0.2) m- (.) as I was talking he (0.4) sort of- 
10  (0.3)
11  I10: Right_ 
12  W10: (°°Put it in,°°)
13  I10: And how long was his penis inside your mouth for would you 14 say,
14  (0.9) 
15  W10: Maybe like, erm .hhh (0.3) like three or four minutes 
16       (0.2) maybe?
17  (0.4)
18  I10: Okay. (1.8) Okay, (0.5) And (0.3) what was (1.2) you know ha- 
19       (0.2) was he motion (.) motion at all during this time, 
20       anything you know, what were you- what you- what was his body 
21       doing at that time_
22  (.)
23 W10: Erm: (1.5) er: (0.6) I didn’t really >notice °his°< (0.5) 
24       >body doing anything< I just (0.3) remember his (0.2) head 
25       (0.5) >sort of< (0.3) not his head his hands sorry (.) moving 
26       my head (0.3) .shihh em (0.3) back and forth,

The interviewer uses a grammar of nonagency throughout [38,60], choosing formulations such as ‘his penis entered your mouth’ (line 2). No autonomy or responsibility is assigned to the suspect for what happens to his penis. The verb (entered) describes movement past a threshold with no indication of the source of motion. In contrast, the emphasised possessive ‘your’ in relation to the complainant’s mouth foregrounds her connection to (and responsibility for) the ‘entered space’, thus implicating her capability to permit or refuse entrance without invoking the suspect’s comparable control over his penis’ entry. After some turn-initial disfluency, possibly marking a problem parsing the clunky non-agentic question design, the complainant’s response (lines 6–12) orients pointedly not just to the suspect’s agency for his actions but also to his agency in attempting to (physically) silence her verbal non-consent to his actions (as I was talking he sort of put it in). The complainant clarifies verbal non-consent twice and uses active grammar to describe what the suspect did with his penis.

During the complainant’s response (lines 6–12), the interviewer provides three minimal receipts (lines 5, 7, and 11). The first treats ‘literally straightaway’ as a sufficient answer; the subsequent two (7 and 11) treat the answer as complete and mark the hearable in-progress account of how the suspect’s precipitous action interrupted her attempts to refuse consent as being irrelevant to the question that was asked. In the interviewer’s next turn, the turn-initial connective ‘and’ links ‘and how long was his penis inside your mouth for would you say’ (lines 13–14) to his previous as a chained question, furthering the sense in which suspect’s active role and the complainant’s verbal non-consent is disregarded. When responding to this question, the complainant is succinct and does not challenge the non-agentic question-formulation again, though she does not use it herself (lines 15–16).

The interviewer persists with non-agentic suspect constructions for his next question as well, even when doing so requires multiple self-initiated repairs to achieve (lines 18–21). This highlights the non-normative nature of this type of question and the discursive effort required to elide the suspect’s agency behind ‘what was his body doing at that time’ (lines 20–21). Repairs like these are a powerful marker to both interlocutors and analysts about what the speaker treats as sufficiently important to justify disrupting the progressivity of the conversation to correct or include [61]. In response to the interviewer’s persistence, the complainant begins to adopt passive constructions of the suspect’s actions. However, her talk is marked with reluctance and discomfort. For example, there are markedly faster and quieter portions of talk, pauses, fillers such as ‘er’, and self-corrections (lines 23–26).

The interviewer’s persistence with non-agentic language appears to privilege and protect the suspect by removing them from the talk about the assault. It reveals the institutional power of the interviewer as, over successive turns-as-talk, the complainant acquiesces and begins to use the same formulations. Thus, the narrative of the assault as it is first produced within the police record begins to minimise and remove the suspect’s role. We have shown here how a police interviewer can exercise their discursive and institutional power to erase Mens Rea from the suspect and remove his thinking and actions from the situation. The implications of this shift for the case’s progression through the wider CJS appear only to benefit the suspect [62].

The complainant’s observable discomfort is accentuated by further power asymmetries in the interview—the interviewer is male, and the complainant is young and vulnerable (she discloses her autism diagnosis early in the interview). From a position of social, interactional, and institutional power, the interviewer uses complicated and opaque grammatical formulations that recurrently and implicitly disadvantage the complainant’s account in favour of protecting the suspect. It is in this sense that the interviewer’s actions could be described as aligning with the wider patriarchal ideology of the institution and of society.

While not a conscious aim, the adversarial justice system in England and Wales works on the basis of the presumption of innocence, which is indeed fundamental to justice. However, in terms of rape, which is a heavily gendered crime and often has few if any witnesses, this adversarialism can often be detrimental to complainants, often placing responsibility onto the complainant while exonerating and erasing the suspect—in this case, using agentless passives. This is a pattern identified by the End Violence Against Women Coalition (EVAW, 2019) [2] amongst others and which is also reflected in, and reflects, wider societal practices such as media reporting of rape [39].

### 3.3. Partner Profile

Misunderstanding constructions become yet more complex when examining partner rape cases and often further relate to communicating non-consent properly, even when the complainant has affirmed and reaffirmed her verbal non-consent. Indeed, as the relationship between victim and perpetrator becomes closer, the extremity of the use of language to challenge victimhood increases, as seen in the final two extracts. Extract 5 is an interview with a complainant who was in an abusive relationship with the suspect, who had a history of sexual, physical, and emotional violence against the complainant. This was ongoing almost up to the interview date, and the complainant’s description of the incidents suggests she was coerced. An appropriate adult was present, indicating she is vulnerable. The talk is about the complainant’s understanding of rape and consent, and the suspect’s understanding of non-consent.

**Extract** **5:Int 05:** *And are we sure that L knew that you don’t want to have sex?*


1   IO5: Okay. .hhhh So what’s your understanding of rape now_ (0.2) 
2        Wha what d’you think rape is now_
3   (0.9)
4   IE5: Literally if a wo:man says n:o (0.6) and then (0.3) then a 
5        man’s got obv’sly take that as a no or othe:rwise it’s (0.9) 
6        obv’sly classed as rape,
7   IO5: °Yeah° (0.7) °That’s it°, .hhh And that man’s got to know that 
8        you mean no, 
9   IE5: M [: : mm. ]
10  IO5:   [and that you d]on’t want sex. .hhh An:d (0.6) are we sh- 
11       are we- sure that **L1***((suspect))* knew that you don’t want to have 
12       sex.
13  (0.9) 
14  IE5: I think he knows that. .hh He knows but (1.5) he’s >one uh 
15       th<em people who will not- he won’t take no f’r an answer off 
16       anybody,
17  IO5: °Okay°, .hhh So (0.8) we’ve briefly spoke abou- >Is there 
18       anythin< else that you can think abo:ut, Because obviously I 
19       appre:ciate when you’re in a relationship and you’re sayi:ng 
20       .hhh what- You know- Someti:mes (.) what’s in our he:ad and 
21       w- an you d- an you’re thinkin i don’t want to have sex, .hh 
22       That person that you’re having sex with in- **L1** in this case 
23       always wanting sex, .hh he’s got to know that you don’t want 
24       to,
25  (5.6)
26  IE5: N:o, 
27  (7.4) 
28  IE5: It’s just he ne:ver knows he-he (0.3) he always (1.1) no 
29       matter f’r how much I say no it doesn’t go >through he’ll< 
30       just carry o:n,
31  IO5: Okay


The interviewer initially asks the complainant what her current understanding of rape is (lines 1–2). It is worth noting that Hohl and Stanko (2015) [18] found that the likelihood of case dropout rose significantly if investigators judged a complainant had a ‘lack of understanding of consent’. The complainant’s definition is general but broadly correct in line with the legal definition and traditional ‘no means no’ understandings, although not with the contemporary affirmative consent model, which is a more up-to-date understanding. She had previously expressed that she repeatedly verbally refused (withheld consent) and was unwilling, an important nuance in terms of consent, as someone can wish to have sex but not give explicit consent (or not be willing, yet give consent, potentially due to coercion) [34]. The interviewer orients to this definition and continues with ‘no means no’, in line with other interviewers’ models of consent in the data. She caveats and modifies the complainant’s definition to include (and emphatically stresses) her responsibility to ensure that her refusal was recognised by the suspect—‘and that man’s got to know that you mean no’ (lines 7–12). The interviewer then asks the complainant, ‘Are we sh- are we- sure that L1 knew that you don’t want to have sex?’ (lines 11–12). This formulation shifts the suspect’s role into the past tense, implying that the suspect’s actions are over, thus backgrounding and reducing any current responsibility. However, when dealing with the complainant, the wording is in the present tense, implying that some responsibility still rests upon her for communicating non-consent. Thus, in the context of defining rape, the key question becomes not what the suspect did but whether there is collective and enduring certainty about the suspect’s knowledge of the complainant’s non-consent (‘we’—either among the police or including the complainant). Lines 7–12 convey not only the interviewer’s doubt in the complainant’s account and their understanding of rape but also invoke stereotypes such as ‘secretly wanting it’ or ‘he didn’t mean to’.

Between lines 17–24 the interviewer speaks with considerable disfluency and multiple re-starts displaying some interactional trouble. This manages the interactional and social delicacy of expressing disbelief in an interlocutor’s statements whilst nevertheless enabling the interviewer to do just that, suggesting that the complainant did not, in fact, consent verbally and instead just thought it. This is achieved through several discursive moves: The interviewer normalises not communicating non-consent through the plural you (‘when you’re in a relationship and you’re sayi:ng’) and appeals to common knowledge (‘you know-‘). She shifts footing to the inclusive ‘what’s in our he:ad’ to treat ‘thinkin i don’t want to have sex’ as acceptable and relatable [55]. The interviewer conveys that thinking rather than verbalising sexual refusal is particularly understandable in circumstances that match the complainant’s (e.g., ‘in a relationship’ and ‘that person that you’re having sex with in-L1 in this case always wanting sex’. In so doing, the interviewer avoids directly asserting disbelief but nevertheless expresses a clear implication that the complainant did not verbalise non-consent and cannot claim that the suspect knew she did not want sex. She ends her extended turn by reiterating that ‘he’s got to know that you don’t want to’ in order for the encounter to constitute rape.

The lack of a direct accusation makes it interactionally more complicated for the complainant to resist the implication [63] and might help account for the extremely long pause before she responds. Her simple response (‘No’) does wholly reject the implication, but the interviewer’s subsequent lack of response (line 27) treats her rejection as incomplete. She then goes on to dismiss her actions in the moment as inconsequential when faced with the suspect’s generalised pattern of behaviour to disregard anybody else’s answers (‘no matter f’r how much I say no it doesn’t go >through he’ll< just carry o:n)’. This builds on her earlier construction of the suspect as dispositionally ‘one uh them people who ... won’t take no for an answer off anybody’ (lines 14–15) [64] and challenges the discursive construction that non-consent relies on the complainant being certain of the suspect’s knowledge of her sexual refusal.

This extract reveals how the interviewer invoked and normalised stereotypes around suspects ‘not realising’ they were having non-consensual sex, which specifically cast doubt on the complainant’s version and constructed her as culpable for any consent misunderstandings. It shows how the complainant’s attempts to resist the interviewer’s version of events are constrained and limited through the interviewer’s interactional and institutional power to control the agenda of the questions asked and to arbitrate ‘correct’ definitions of legal concepts like consent. Given the complainant’s profile as a vulnerable complainant of partner rape, the interviewer is likely aligning with wider institutional and societal ideologies and practices around real rape complainants and credibility.

Extract 6 relies on a grammar of nonagency and some passive talk to obscure suspect autonomy and agency. This is a violent partner rape, and in addition to the non-agentic language, neutralising and non-violent language is used. The line of questioning pertains to positioning and exact facts of the account.

**Extract** **6:Int 09** *So his penis went into your vagina.*


1   IO9: Oka:y*?* (0.2) Er: and you said he put hi:s (.) er dick inside 
2        you, 
3   IE9: .HHHH
4   IO9: Inside you where.
5   IE9: Inside my vagi:na,
6   IO9: Okay? [So his ] pe:nis= (0.2) 
7   IE9:       [.hhSHih]
8   IO9: =went into your vagina.= 
9   IE9: hYeah,
10  IO9: Yeah*?* [Oka:y*?*]
11  IE9:       [.SHIH ]
12  (0.3)
13  IO9: Tell me about the: (0.3) posi:tioning in relation to where 
14       you were lay (.) [or- or standing_ ]
15  IE9:                 [>I was< on my back, hhu.hh]
16  IO9: You were on your ˆbackˆ o:kay*?*
17  IE9: .SHHIH=
18  IO9: =Er:m (0.3) and he’s taken your pyjamas and your pants off 
19       (.) yeah*?*
20  IE9: >.SHUHH< Yeah_
21  IO9: An:d (0.3) and then he: (0.7) 
22       what climbs on top of you,[or,]
23  IE9:                           [he ]climbs on top,
24  IO9: [yeah*?*] (0.6) [Okay, ] 
25  IE9: [.SHIH]     [tkhuhh]
26  IO9: And he’s holding your shoulders [down,]
27  IE9:                                  [he’s ] got me pinned down so 
28       I can’t move he had (.) his hands like that on me so I 
29       couldn’t move my arms or no:thing, hhh [.shIH]
30  IO9:                                         [Okay?] 
31  (0.7) 
32  IE9: Euhh [ .hhh ]
33  IO9:      [And what’s] being said.
34  IE9: .shUh (.) He was saying I’m just gonna be his dirty slag and 
35       his bitch (.) I’ll do what he (.) do what he says, .hshihhh 
36       (0.2) And I couldn’t say nothing cause I had a sock in my 
37       mouth, .shih


The interviewer reformulates the complainant’s description of what the suspect did to her (‘you said he put his er dick inside you’, lines 1–2) into a more neutral, agentless, and passive version, ‘so his penis went into your vagina’ (lines 6–8) [38]. ABE interviews prefer medical terms, which might contribute to the replacement of more colloquial terminology. However, the reformulation goes further than anatomical clarification and exemplifies the pattern shown earlier in our analysis to minimise suspect agency in the descriptions of events (changing ‘he put his dick’ to ‘his penis went’).

The interviewer recurrently uses neutral language in place of more violent words. For example, earlier in the interview, the complainant had said, ‘he’s got me pinned down’; here, the interviewer reformulates that into ‘he’s holding your shoulders down’ (line 26). The complainant resists the neutral characterisation of the grip as ‘holding’ her by reasserting her original formulation of ‘pinned down’ (line 27). She then extends her description to include the physical consequences of his grip, again repeated from earlier in the interview, that she could not move her arms. Immobilisation is more consistent with pinning than holding, so by including that detail, the complainant provides evidence to support her word and reject the interviewer’s version. An Italian study examining courtroom questioning reflects this pattern, finding that the defence replaced terms such as ‘violence’ or conflict’ with words such as ‘squabble’ or ‘predicament’ [65]. The police interviewers in our data are not defence attorneys. The interactional goal of their role in the interview is to gather evidence, not defend the suspect. This makes it all the more striking to find similar discursive resources being mobilised between the two contexts.

As the extract continues, the interviewer uses more agentless talk: ‘and what’s being said.’ (line 33). Isolated from the full context of the interview, this question could appear reasonable—the complainant could be saying no, or the suspect may be speaking. However, the complainant had already established earlier in the interview that she had been gagged, preventing speech. To ignore that context by creating an ambiguity about who could have spoken here diminishes the suspect’s power to silence the complainant and places equal emphasis on her as a participant in the rape. The complainant reaffirms her inability to speak in her response and confirms what the suspect was saying to her (lines 34–37).

In choosing to ascribe equal potential for speech to both the suspect and complainant, the interviewer pulls focus from the suspect and uses it to exaggerate the complainant’s freedom of action during the rape. Here, the interviewer exercises their discursive and institutional power to control what gets scrutinised during the police investigation. A systematic interactional pattern of minimising the suspect’s role and maximising the complainant’s role opens the door for an overbearing institutional focus on the complainant and an associated disregard and minimisation of the suspect’s role with potentially profound judicial implications.

## 4. Discussion

Our analysis demonstrated that interviewers could use their institutional and interactional authority over complainants to construct suspects as someone who misunderstood or as a set of actions with hidden or obscured intentions. This reflects wider institutional and societal ideologies of sexual violence, a point reinforced by McMillan (2018) [66], who noted patterns of disbelief and cynicism about complainants in interviews with police officers that are related to a rigid hierarchy and a culture of hegemonic masculinity where women’s bodies and accounts are called into question to protect the presumption of innocence of the suspects, who are predominantly male and thus members of the patriarchal ingroup [66,67,68].

There is an ongoing construction of rape suspects as having missed or misunderstood non-consent. This is related to what the complainant did or did not say or do, thereby causing or failing to prevent the misunderstanding, and is a form of covert complainant-blaming. The responsibility of making clear their non-consent and ‘doing everything they can’ is placed on the complainant, despite varying reactions and levels of resistance for complainants—some freeze and feel unable to move [69], which may cause further blame to be placed on them, particularly where higher levels of RMA already exist [70].

The complainants had all verbally expressed non-consent beforehand but were often still asked about physical resistance, visible signs of distress, and whether they believed the suspect knew they did not want sex. This was more common the more intimately the complainant and suspect knew each other, with questions in partner rapes often centring on understanding consent and being certain that the complainant was clear towards the suspect, which falls in line with the legal definition of rape and outdated models of consent, which, while the data were pre-#MeToo, can still be seen in other recent research [9]. This responsibility shifting indicates both individual and institutional disbelief in rape accounts, potentially due to the presumption of innocence and adversarialism inherent to the justice system. This becomes stronger the more likely cases are to be ‘complex’ rapes—partner or acquaintance profiles, for example. This supports previous evidence that complainants of stranger rape are the least blamed, complainants of marital rape the most, with acquaintance rape complainants falling in between [71,72,73].

Passive, agentless, and violence-neutralising language was commonplace when speaking about the suspects. For stranger rapes, these instances were frequent and often centred on body parts and suspect actions towards the complainant. For acquaintance and partner rapes, these were less frequent, but when they did occur, they were more likely to be in the form of neutralising language such as ‘have sex with’ or ‘held down’. Similarly to the misunderstanding construction, this shifts responsibility away from the suspect by removing attributions of intent and thoughts and rendering body parts autonomous, and in some cases, acting as a way of expressing disbelief. This construction is also in line with previous research—agentless passives are used in the media [60,74] and in legal settings [38,75]. Obscuring suspect agency with this grammatical device affects attributions of responsibility and harm [74,76], which could be detrimental in the legal context. Notably, Ehrlich’s (2003) [38] examples of agentless passives were of the suspect using them, with the effect of diffusing responsibility and creating uncertainty. This makes it striking that the police interviewers are orientated to the same structures here, with the same effects.

### 4.1. Implications

The interviewers’ constructions throughout the dataset (particularly those that had the effect of shifting responsibility from suspect to complainant) often distressed and confused the complainant. Perceived harm and Mens Rea were diminished through suggestions that the complainant could have done more to avoid the rape. The interviewer appeared frequently ideologically aligned with the institution, displaying their particular mental models of rape through the questions asked and the way questions are formulated [66]. This is likely due to factors such as the general patriarchal values of society perpetuating social representations of rape throughout institutions. Also, the adversarial justice system’s reliance on ‘innocent until proven guilty’ potentially influences social representations in ways which emphasise the innocence of the defendant to the detriment of the integrity and credibility of the complainant [77]. Even if individual interviewers’ personal beliefs are not rape-supportive or high in rape stereotype acceptance, several previous authors have noted that the presence of rape stereotypes in their respective data was likely symptomatic of a larger problem [26,27]. Indeed, regardless of any intent to build a strong case for the complainant, the way questions are asked is still alienating complainants from the justice process, potentially contributing towards feelings of blame and secondary victimisation. Thus, interviewers are still aligning themselves with the overall ideological goals of the police force and the CPS. Interviews are being used to inform decisions about the next steps in investigations, and the real rapist stereotypes inherent within them, unconscious or not, create bias in decision-making. Should the case go on to court, defence lawyers may use the constructions and stereotypes within the interview as evidence (or lack thereof), possibly harming the complainant’s credibility and affecting jury decision-making. This is especially true for acquaintance and partner rapes, where there are more real rapist stereotypes and more misunderstanding constructions. This could be a consequential contributor to the ‘vicious cycle of attrition’ in the UK [4], causing many rape complainants to fall through the cracks.

In terms of theoretical implications, this study took a novel methodological approach. We drew from micro-level conversation analysis and discursive psychology to explore conversational details at a level typically left out of legal transcripts (such as timing, overlap, emphasis, and speech disruptions). We placed these features within the wider context of the interview participants, power and asymmetry, and social representations of rape via the macro-level socio-cognitive CDA. This displays the utility of taking a pluralistic approach to analysing legal transcripts and supports van Dijk’s (2001) [49] proposal that CDA should be diverse and multidisciplinary suggesting more research should be conducted within the CJS using this approach.

### 4.2. Recommendations

The findings suggest ambitious recommendations for policy and practice are necessary. As Operation Soteria Bluestone (2023) [78] highlighted, it is essential that specialised teams with expert knowledge handle rape and other sexual offences. This expertise is crucial in providing complainants with the necessary support, including specialised interviewing techniques that consider the dynamics of coercion and control exerted by suspects, which interviewing officers often do not understand or pay attention to. This may reduce reliance on misunderstanding narratives and agentless passive talk to background the suspect. It may facilitate a shift towards suspect-focused investigations and interviews, emphasising the behaviour and actions of the suspect rather than questioning the credibility and actions of the complainant. Some steps are already being taken towards this, with guidance on complainant-blaming for police [79].

To help reduce rape stereotype acceptance and use, widespread, standardised, and longitudinally tested training with an evidence-based theoretical framework is necessary. Murphy and Hine (2019) [8] suggested utilising a cognitive framework in police training to improve interviewing skills in terms of increasing clarity and empathy for complainants [80] and address the mechanisms behind attitude change, stereotypes, and prejudice. Social Representations Theory would be a good fit for this cognitive framework, providing an understanding of how stereotypes are underpinned and perpetuated. It has been found that interviewing skill with rape complainants is better with lower rape myth acceptance [80]; thus, it is important that any training encompasses the multiple factors surrounding police rape stereotype acceptance. This includes possible unconscious biases caused by sexism, traditional views on women, or ‘kinds’ of complainants [81,82,83,84], and allows for understanding good investigative decision-making without over-reliance on cognitive ‘shortcuts’ and social representations of rape [85].

Finally, any change made to practice must be folded into a system-level change across the CJS as the entire ecosystem is interdependent: this means jury-based education, equitable prosecution policy, and good practice in law and policing.

### 4.3. Limitations and Future Research Directions

The interview sample consisted of White rape complainants only. Given that Black and Asian women are at highest risk of sexual victimisation, it is necessary to conduct sensitive, Black-centred co-produced research to further understand cultural differences and issues in police interviewing of rape complainants.

No case outcome information was available—i.e., whether police had decided to take no further action, take the case forward, or whether the complainant withdrew at this or a future stage. Outcome data in similar future work would add further understanding to the impact of interviewing and stereotype use on the complainant. For example, Pipe et al. (2013) [82] found in a child interview study that there was a possible link between following interview protocol and better case progression, i.e., more guilty verdicts and charges.

Exploring ABE compliance in future research would be useful. A content analysis, along with analysing examples of good or poor practice and comparing these with the presence or quantity of rape stereotypes, could provide insight into how or whether ABE compliance mediates rape stereotype use.

Finally, due to the sensitivity of the data in this study, only the first author was allowed into the police facility to transcribe the interviews. This could undermine data trustworthiness; however, transcription training sessions, data sessions, and regular meetings with the co-authors mitigated this, and the team felt confident in the credibility and trustworthiness of the transcripts and analyses.

## 5. Conclusions

We found an ongoing pattern of shifting responsibility away from the suspect and onto the complainant. Interviewers constructed him as having misunderstood or missed non-consent or by using passive-agentless talk that obscured his autonomy and removed his agency. There was a secondary effect of expressing disbelief in the complainants’ accounts. This is concerning and is a possible contributor to the high attrition rate at this investigative stage. Recommendations for policy and practice include specialist knowledge and teams for handling rape cases, training with a cognitive framework to address underlying biases, beliefs, and assumptions, as well as understanding stereotypes. This is alongside work to improve rape case responses within the CJS as a whole-systems approach, to reduce decision-making shortcuts within the police.

## Figures and Tables

**Table 1:** Definitions of **complainant**-**suspect** relationships.

Relationship	Definition
*Stranger*	Offences ‘where the complainant and suspect are stranger or unknown to each other’ [50]. Very limited legitimate contact or previous non-legitimate contact is included in this definition. Although stranger rapes are considered the most common type, in reality, only approximately 16% of rapes are by strangers [51]. This is reflected in the current data, in which only three interviews, two of which were used in the analysis, were stranger rapes.
*Acquaintance*	Offences where ‘the complainant and suspect are known to each other but have not had a previous sexual relationship’ [50]. Three types differing in closeness to the complainant were identified in the current data: Where they have known each other for just a few days, where the suspect is a ‘friend of a friend’, and where the suspect is a friend of the complainant.
*Partner*	‘Offences committed by people who are, or have been, intimate partners’ [52]. The nature of the rapes in the study’s interview data either includes coercive control in order to commit sexual violence or physical and sexual violence. The former is more common than the latter in the data, although sometimes the two are combined.

**Table 2:** Jefferson transcription conventions.

Transcription Feature	Meaning
w[ord]	
[wor]ds	Overlapping talk
word=words	Latched utterances
(0.5), (2.4)	Longer pause in seconds
(.)	Micropause, considered >0.2 s
Wo:rd	Extension of the sound or syllable
Wo::rd	A more prolonged stretch
Wo:rd	Downwards intonation in the middle of a word before rising again at the end
Wo:rd	Upwards intonation in the middle of a word before falling again at the end
.	Falling final intonation
,	Continuing intonation
?	Rising final intonation
*?*	Medium final intonation
WORD/WOrd	Loud talk
Underline, underline	Emphasis on all or part of a word
°word°	Passage of talk that is quieter than surrounding talk
<word>	Passage of talk that is slower than surrounding talk
>word<	Passage of talk that is faster than surrounding talk.
**^word^**	**Passage of talk that is higher in pitch than surrounding talk.**
Hh	Audible aspirations
.hh	Audible inhalations
(hh)	Laughter within a word
.huhh huh (huh)	Crying or sobbing
.shuhh/.shih	Sniffing
((gesture))	Transcriber’s comments

## Data Availability

The data presented in this study are available on request from the corresponding author due to the distressing nature of the materials, and while the data is fully anonymised, it is preferred to limit access to further protect the privacy and anonymity of the complainants as far as possible.
